# A Model for Dry Deposition of Atmospheric Micro- and Nanoplastic Fibers

**DOI:** 10.1021/acs.estlett.6c00165

**Published:** 2026-04-29

**Authors:** Hosein Foroutan

**Affiliations:** Department of Civil and Environmental Engineering, 122386 Virginia Tech, Blacksburg, Virginia 24061, United States

**Keywords:** microplastics, nanoplastics, dry deposition, atmospheric modeling, fibers, aerosol

## Abstract

Atmospheric microplastics and nanoplastics (MNPs), particularly fibers, are emerging contaminants with complex deposition dynamics that remain poorly understood. Here, a mechanistic dry deposition model is developed for MNP fibers, integrating gravitational settling, Brownian diffusion, impaction, and interception. The model incorporates fiber-specific drag corrections and shape-dependent diffusivity to estimate total deposition velocity across a range of particle lengths and aspect ratios. Results show that total deposition velocity deviates significantly from gravitational settling alone, especially for submicron fibers. Global simulations reveal strong spatial and seasonal variability driven by land cover and turbulence. Atmospheric lifetime estimates based on dry deposition indicate that micro – nano transition fibers (∼ 1 μm) persist longest in the boundary layer, with dry-deposition lifetimes exceeding four months (∼ 2,900 h). This framework supports the integration of MNP fibers into existing aerosol dry-deposition schemes in regional and global atmospheric models and improves predictions of their environmental fate.

## Graphical abstract

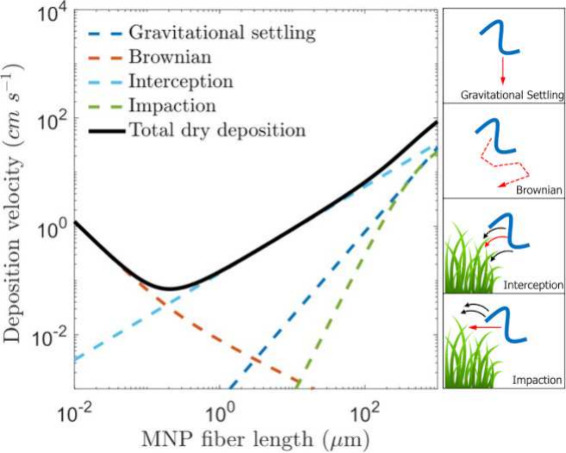

## Introduction

Atmospheric microplastics and nanoplastics (MNPs) have emerged as a pervasive form of environmental pollution with significant implications for ecosystems and human health.^1–6^ Among the various shapes of plastic particles detected in the atmosphere, fibers consistently dominate,^4,7^ underscoring their ubiquity and potential importance in atmospheric processes.

Despite their prevalence, mechanisms governing emission, transport, and deposition of MNP fibers remain poorly understood. Current research employs field sampling with back-trajectory analysis^8–11^ and atmospheric modeling,^12–17^ both requiring realistic representation of dry deposition—a major atmospheric removal pathway.^18^

The dry deposition of atmospheric particles is a complex process influenced by factors such as particle size, shape, density, and environmental conditions.^19^ Early efforts to quantify MNP deposition often relied on Stokes’ law, treating gravitational settling as the primary mechanism.^9^ However, this approach oversimplifies the behavior of elongated MNP fibers, which exhibit aerodynamic properties distinct from spherical particles. Recent studies have developed parametrizations to account for the settling velocities of these fibers, providing a more nuanced understanding of their deposition dynamics.^15,20,21^ More recently, physically based formulations have been proposed to describe atmospheric deposition of microplastics, including fiber-shaped particles, within generalized dry and wet deposition frameworks.^22^ Nonetheless, other critical deposition processes—such as Brownian diffusion, impaction, and interception—are typically treated without fiber-specific parametrizations and are therefore effectively neglected for elongated MNP fibers. This gap likely reflects a historical focus on larger plastic fibers and the challenges in detecting and characterizing smaller MNPs.^23^ As detection methods improve and the potential health risks of smaller MNP fibers become more evident, the need for a comprehensive understanding of their dry deposition grows increasingly urgent. To fully integrate MNP fibers into atmospheric models, a physics-based framework consistent with existing aerosol schemes is needed. Such representation remains largely absent in current simulations.^12,15,24^

Here, I address this critical gap by presenting a physics-based model for the dry deposition of MNP fibers. The proposed approach builds on existing knowledge of aerosol dry deposition while incorporating the unique physical properties of fibers, offering a consistent framework that can be seamlessly integrated into atmospheric models spanning local to global scales. The spatiotemporal changes in deposition velocity of MNP fibers and global atmospheric residence time against dry deposition were evaluated using this model and are discussed in the context of fiber properties as well as atmospheric and land conditions.

## Theory

Dry deposition of atmospheric aerosols refers to the removal of particles from the air onto the Earth’s surface via gravitational settling, Brownian diffusion, interception, and impaction.^19^ The relative importance of these mechanisms depends on the effective size of the particles—a factor that becomes especially critical when representing nonspherical MNP fibers. Gravitational settling dominates for larger particles, whereas Brownian diffusion is the primary removal pathway for ultrafine aerosols.

Despite decades of research, substantial gaps remain in our mechanistic understanding of aerosol dry deposition, regardless of particle shape. As a result, a range of parametrizations have been developed and implemented in regional to global-scale atmospheric models.^25–30^ The selection of a dry deposition scheme often involves a trade-off between physical complexity and computational efficiency.

In this work, I started from the dry deposition parametrization of Emerson et al.^28^—which refines the widely used Zhang et al.^25^ model using more recent field measurements—and developed fiber-specific corrections for MNPs. This model describes size-dependent dry deposition velocity as a function of gravitational settling velocity *V*_*g*_ and two parallel resistances—an aerodynamic resistance *R*_*a*_ and a surface resistance *R*_*s*_:^28^

1 $$V_{d}=V_{g}+\frac{1}{R_{a}+R_{s}}$$

The surface resistance term accounts for particle losses due to three processes: Brownian diffusion, impaction, and interception.

2 $$R_{s}=\frac{1}{\epsilon u_{*}\left(E_{\mathrm{br}}+E_{\mathrm{im}}+E_{\mathrm{in}}\right)R_{1}}$$

These processes are quantified using collection efficiencies, defined as

3 $$E_{\mathrm{br}}=0.2\cdot {Sc}^{-2/3}$$

4 $$E_{\mathrm{im}}=0.4\cdot {\left(\frac{St}{\alpha +St}\right)}^{1.7}$$

5 $$E_{\mathrm{in}}=2.5\cdot {\left(\frac{d_{p}}{A}\right)}^{0.8}$$

Here, *Sc* is the Schmidt number, *St* is the Stokes number, and *d*_*p*_ is the particle diameter. α and *A* represent characteristics of the deposition surface and are typically specified in models based on land use type. The rebound correction factor was set to *R*_1_ = 1, assuming no particle bounce upon contact, consistent with adhesion-dominated microparticle impact theory at low normal velocities.^31,32^ All three collection efficiencies—Brownian diffusion (*E*_br_), impaction (*E*_im_), and interception (*E*_in_)—are either explicitly or implicitly size-dependent, with their magnitudes strongly governed by particle dimensions and dynamics.

To represent the effective size of atmospheric MNP fibers across these processes, I use the equivalent diameter *d*_*eq*_. For generalizability and potential implementation in large-scale models, MNP fibers are assumed to be cylindrical with a length *l* and diameter *a*, defined by an aspect ratio β = *l*/*a*. Fibers are treated as rigid cylinders defined by length, aspect ratio, and density. Mechanical and microstructural properties such as flexibility, surface roughness, or core–shell morphologies are not considered, as these are generally unavailable in large-scale models and would substantially increase complexity. The present formulation therefore does not explicitly account for deformation, bending, orientation dynamics, or microstructural heterogeneity associated with real fibers.^33,34^ These effects may influence deposition under weak turbulence or at small scales and represent important directions for future mechanistic and experimental work; however, inclusion of such properties is currently not feasible in large-scale atmospheric models and falls beyond the scope of the present parametrization.

Several studies have examined the gravitational settling velocities of irregularly shaped particles, typically tens of micrometers or larger.^20,35–38^ Although these studies vary in complexity, they commonly introduce a corrected drag coefficient based on an equivalent diameter. Here, I adopt the parametrization by Zhang and Choi,^36^ who developed a drag coefficient based on the Aschenbrenner shape factor for microplastic fibers with Reynolds numbers in the range of 1–300. For cylindrical MNP fibers, the Aschenbrenner shape factor simplifies to the aspect ratio β, and the drag coefficient *C*_*d*_ and equivalent diameter *d*_*eq*_ are^36^

6 $$C_{d}=\frac{58.58\cdot \beta^{0.1936}}{Re^{0.8273}}$$

7 $$d_{eq}={\left(\frac{4l^{2}}{\beta \pi }\right)}^{1/2}$$

Gravitational settling velocity *V*_*g*_ is then calculated iteratively from the balance of gravitational and drag forces. The settling velocities predicted by eq 6 are within 10% of recent laboratory measurements of isolated microplastic fibers in air reported by Reininger et al.^39^ (see their Table 2). Alternative formulations for *V*_*g*_, *C*_*d*_, and *d*_*eq*_ for MNP fibers have also been proposed in the literature,^21,35^ and may serve as alternatives to the Zhang and Choi^36^ scheme.

Very few experiments have focused on nonspherical nanoparticles, where Brownian diffusion dominates.^40,41^ Tian et al.^42^ developed a computational framework for nanofibers (diameters from 1 to 1000 nm, and aspect ratios from 3 to 1000) motion under diffusion, incorporating translation and rotation. The drag force for nanofibers is represented using a translational dyadic tensor, whose diagonal elements for an ellipsoidal particle are

8 $$k_{\mathrm{x̂}\mathrm{x̂}}=k_{ŷŷ}=\frac{16\left(\beta^{2}-1\right)}{\left[\left(2\beta^{2}-3\right)\;\ln\frac{\left(\beta +\sqrt{\beta^{2}-1}\right)}{\sqrt{\beta^{2}-1}}\right]+\beta }$$

9 $$k_{ẑẑ}=\frac{8\left(\beta^{2}-1\right)}{\left[\left(2\beta^{2}-1\right)\;\ln\frac{\left(\beta +\sqrt{\beta^{2}-1}\right)}{\sqrt{\beta^{2}-1}}\right]-\beta }$$

Tian et al.^42^ then proposed the following equivalent diameter under isotropic Brownian diffusion:

10 $$d_{eq}=\frac{a}{\frac{1}{k_{\mathrm{x̂}\mathrm{x̂}}}+\frac{1}{k_{ŷŷ}}+\frac{1}{k_{ẑẑ}}}$$

This equation agrees well with limited experimental data,^40^ and is adopted here to calculate Brownian diffusion coefficients for atmospheric MNP nanofibers by updating the Schmidt number accordingly.

For impaction, the effect of shape is incorporated through the Stokes number *St*, using the gravitational settling velocity *V*_*g*_ updated for MNP fibers. This ensures consistency between settling and impaction formulations.

Interception remains the most uncertain mechanism in aerosol dry deposition,^19^ with limited constraints even for spherical particles. Although interception-equivalent diameters have been proposed for agglomerates,^43,44^ a mechanistic understanding for nonspherical particles is still lacking. In this work, interception efficiency is calculated using the same shape-corrected equivalent diameter derived from Brownian diffusion, ensuring consistency across processes. Varying the interception efficiency by a factor of 5 (consistent with reported uncertainties in aerosol dry deposition^28^) primarily affects the magnitude of deposition velocity while preserving its size dependence and aspect-ratio trends. No additional process-specific modifications to interception are introduced beyond this common diameter correction. Improved constraints on fiber interception remain an important research need given its central role in aerosol removal.^28^

The total dry deposition velocity *V*_*d*_ for atmospheric MNP fibers, like other aerosols, is the sum of contributions from the four processes discussed above (see the Supporting Information document for the full model formulation). Figure 1 shows *V*_*d*_ as a function of fiber length, along with contributions from each removal mechanism. Because size-resolved atmospheric deposition velocity measurements for MNP fibers are not currently available, the predicted deposition velocities should be understood as first-order, physically based estimates that capture the dominant deposition processes rather than fully observation-constrained values. An uncertainty envelope for the proposed fiber dry deposition parametrization—following the ±5× bounding approach of Emerson et al.^28^ and evaluated across multiple land-use types—is provided in the Supporting Information. Sensitivity tests further show that ± 50% variations in fiber density and aspect ratio produce changes in deposition velocity that remain well within this envelope, confirming that fiber-specific parameters do not introduce uncertainty beyond that already associated with surface resistance formulations.

**Figure 1:**
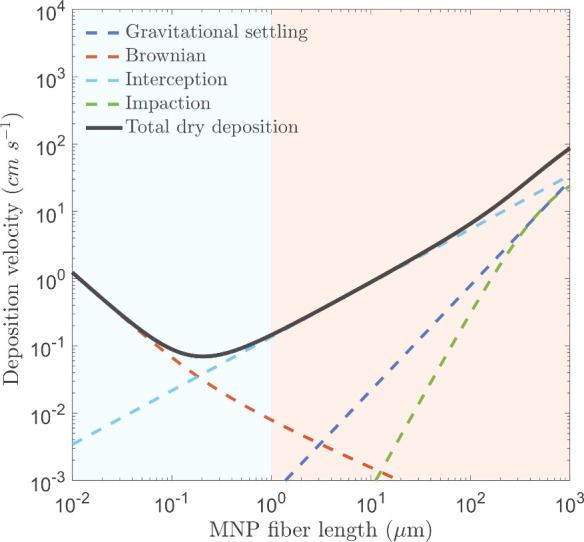
Size-resolved dry deposition velocity (cm s^–1^) of micro- and nanoplastic fibers, showing contributions from Brownian diffusion, interception, impaction, and gravitational settling. The plot assumes deposition onto grass, a friction velocity u_*_ of 0.4 m s^-1^, a plastic density ρ_*p*_ of 1200 kg m^–3^, and fibers with an aspect ratio β of 10. Fibers are assumed not to bounce upon contact with the surface, i.e., *R*_1_ = 1. Shaded regions indicate the range of deposition velocities for nanoplastic fibers (<1 μm, blue) and microplastic fibers (>1 μm, orange). Both axes are on logarithmic scales.

Following Hartmann et al.,^45^ fibers shorter than 1 μm are classified as nanoplastics, while those with lengths between 1 and 1000 μm are microplastics. Overall, *V*_*d*_ for MNP fibers exhibits a minimum near 0.2 μm, with both smaller and larger fibers depositing more efficiently. For nanofibers (<1 μm), Brownian diffusion and interception dominate, with the former increasing rapidly below 0.1 μm. For microfibers, interception and impaction remain important even for large particles (up to 1 mm). Relying solely on *V*_*g*_ for microfiber deposition is therefore misleading. For a representative microfiber of *l* ∼ 300 μm—commonly found in atmospheric fallout^6,14^ —impaction, interception, and gravitational settling contribute approximately 70%, 20%, and 10% of *V*_*d*_, respectively.

## Results and Discussion

The model has implications for variation in dry deposition velocity of MNP fibers across sizes, shapes, and atmospheric and land surface conditions. The relative importance of fiber shape is illustrated in Figure 2(a), where size-resolved deposition velocities are shown for three aspect ratios: 1, 10, and 100. Here, the case β = 1 corresponds to the conventional spherical-aerosol limit and therefore provides a direct benchmark against standard dry deposition schemes that assume spherical particles. Deviations from β = 1 isolate the role of particle shape within an otherwise identical resistance-based framework and quantify the effect of fiber geometry on deposition velocity. As nanofibers smaller than 0.1 μm become more elongated, their deposition velocity increases due to the inverse relationship between Brownian diffusion and fiber aspect ratio. Conversely, for larger microfibers, increasing aspect ratio significantly reduces deposition velocity. For example, a 10 μm fiber with an aspect ratio of 1 (i.e., a sphere) exhibits a deposition velocity nearly an order of magnitude higher than a 10 μm-long microfiber with an aspect ratio of 10. At the upper end of the size spectrum (∼1000 μm), gravitational settling becomes dominant, especially for particles with lower aspect ratios.

**Figure 2:**
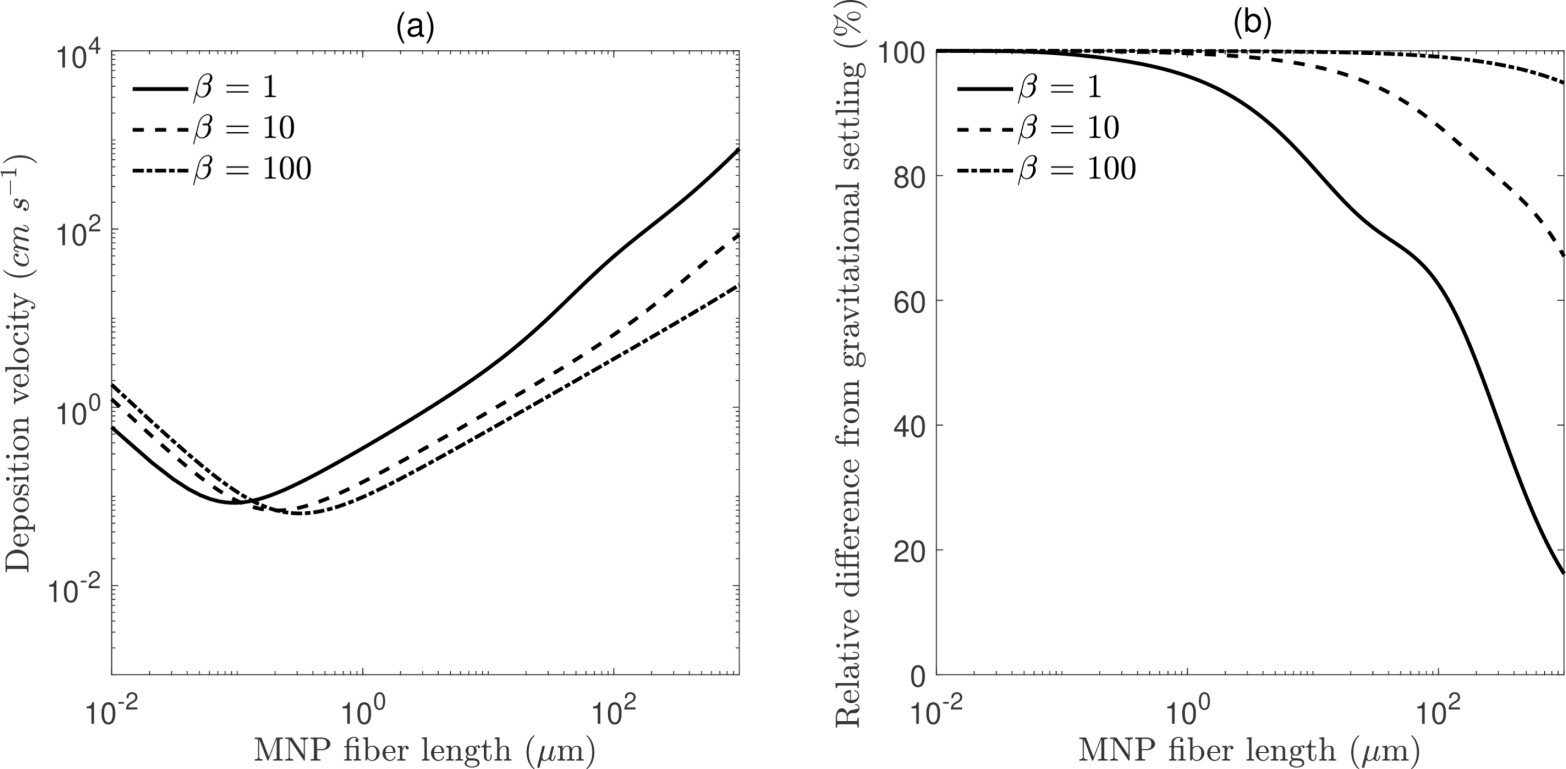
(a) Size-resolved total dry deposition velocity (cm s^–1^) for MNP fibers with aspect ratios of 1, 10, and 100. (b) Percent difference between total deposition velocity and gravitational settling velocity for MNP fibers with varying aspect ratios.

In Figure 2(b), the percent difference is shown between the total deposition velocity (which accounts for all four removal processes) and the gravitational settling velocity alone – a simplified approach used to estimate atmospheric MNP removal. For spherical particles (β = 1), which represent the conventional aerosol limit, this difference decreases rapidly for large particles where gravitational settling dominates, though there remains a 10–20% error due to not accounting for impaction. For smaller particles, even those with near-spherical shape, the total deposition velocity can be approximately 100% higher than gravitational settling alone, consistent with classical dry deposition theory for submicron aerosols. Small nanofibers show a similar pattern, but with consistently high error across a wide range of fiber lengths, underscoring the importance of including all removal processes for elongated MNP fibers. Relying solely on gravitational settling can therefore lead to large biases; for instance, for β = 10, a 300 μm fiber shows an 80% underestimation of dry deposition velocity.

Another important aspect of the current model is that, once all removal processes are accounted for, deposition velocity becomes a function of atmospheric and land surface conditions and is thus spatiotemporally variable—unlike gravitational settling models that assume uniform behavior. Figure 3 shows global maps of dry deposition velocity for a 10 μm fiber with β = 10 during boreal winter (Dec–Feb) and summer (Jun–Aug) of 2020. To generate this figure, friction velocity u_*_ was obtained from the hourly MERRA-2 surface flux dataset M2T1NXFLX,^46^ and land cover was based on the MODIS MCD12C1 product.^47^ Details are provided in the Supporting Information.

**Figure 3:**
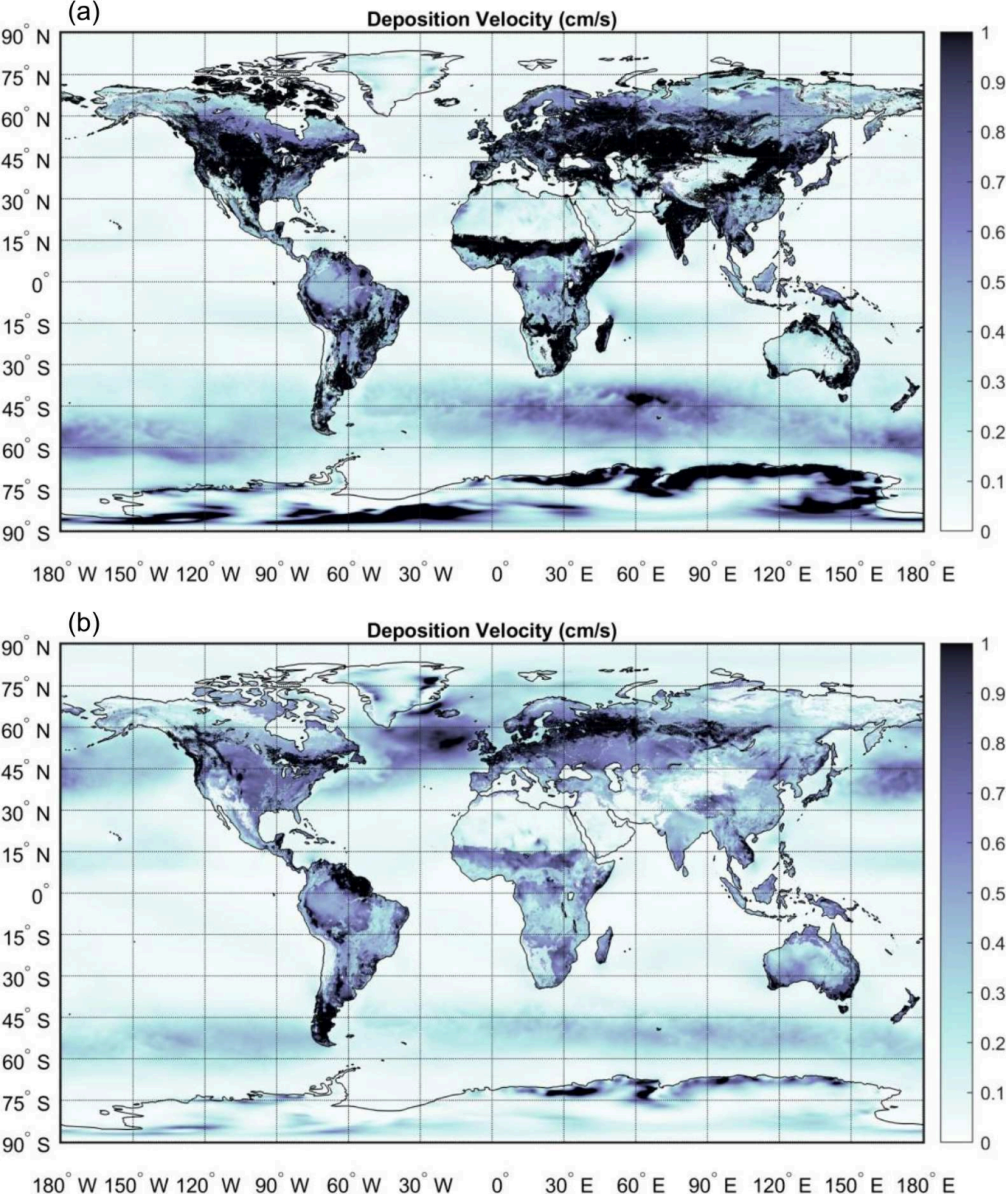
Global seasonal distributions of total dry deposition velocity (cm s^–1^) for a 10 μm microplastic fiber with β = 10 and ρ_*p*_ = 1200 kg m^–3^, derived from simulations for the year 2020 and shown as seasonal means of hourly deposition velocities. Results are shown for boreal summer (Jun–Aug; a) and boreal winter (Dec–Feb; b). Land-surface characterization is based on MODIS land-cover classes.

The global distribution of dry deposition velocity for microplastic fibers shows strong seasonal and regional variability. Summer deposition velocities increase markedly across mid- and high-latitude Northern Hemisphere land areas. In central and eastern North America, Europe, and eastern Asia, *V*_*d*_ commonly reaches 0.7–0.9 cm s^–1^—a 50–80% increase over typical winter values of 0.3–0.5 cm s^–1^. This increase is consistent with enhanced vegetation, stronger turbulence under warmer and more unstable boundary layer conditions, and increased particle interception by vegetative surfaces. High latitudes show pronounced seasonal shifts from winter values below 0.2 cm s^–1^ to summer peaks of 0.6 cm s^–1^, likely reflecting surface thawing and enhanced turbulence. Tropical regions show high deposition year-round with summer enhancement, while oceanic regions maintain lower velocities – typically <0.3 cm s^–1^, consistent with smoother surfaces and the absence of vegetative uptake – with modest seasonal variation. These patterns highlight the spatiotemporal complexity arising from interactions among particle properties, land cover, and atmospheric conditions.

To further quantify atmospheric removal, the atmospheric lifetime τ due to dry deposition was estimated as^19^

11 $$\tau =\frac{\mathrm{PBLH}}{V_{d}}$$

where PBLH is the planetary boundary layer height, taken from MERRA-2 data (see Supporting Information). This formulation isolates the contribution of dry deposition and does not account for wet scavenging or resuspension, in line with the scope of this study.

By systematically varying both fiber length and aspect ratio, the sensitivity of atmospheric persistence to particle geometry was assessed. The results, presented in Figure 4, show that atmospheric lifetime decreases substantially with increasing fiber length, consistent with stronger gravitational settling and surface interaction for larger particles. For fibers with an intermediate aspect ratio (β = 10), lifetime declines from approximately 2,900 h at 1 μm to around 3 h at 100 μm. At fixed lengths, increasing aspect ratio considerably extends atmospheric lifetime. For instance, a 10 μm fiber exhibits a lifetime of about 29 h at β = 1, 295 h at β = 10, and over 1,100 h at β = 100. This pattern reflects the reduced effective deposition velocity of elongated fibers, which settle more slowly and are less likely to interact with surfaces.

**Figure 4:**
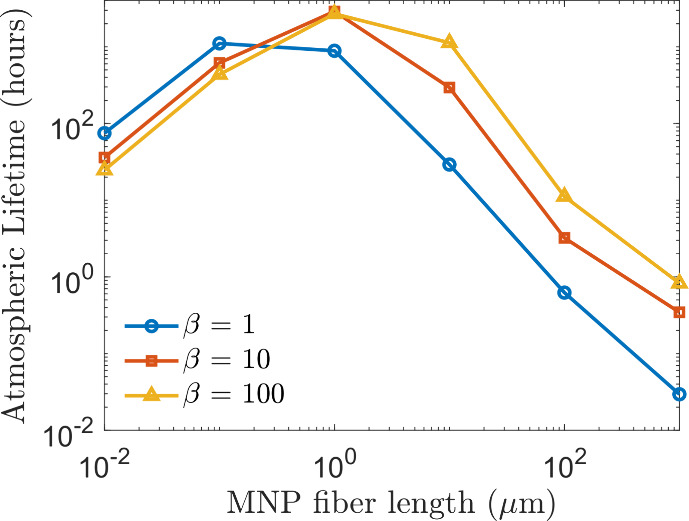
Atmospheric lifetime (hours) of MNP fibers against dry deposition as a function of length and aspect ratio, based on global annual averages of hourly simulations. Both axes are on logarithmic scales.

Interestingly, for very small nanoplastic fibers (e.g., 0.1 μm), the effect of aspect ratio is reversed. At this scale, increasing elongation leads to shorter lifetimes due to the inverse relationship between Brownian diffusivity and aspect ratio. For example, lifetime decreases from around 1,100 h for a spherical particle (β = 1) to roughly 600 h for an elongated fiber (β = 10). Overall, plastic fibers at the micro – nano transition, particularly those around 1 μm in length, exhibit the longest atmospheric lifetimes against dry deposition—exceeding four months (2,900 h) under typical boundary layer conditions.

In closing, this study presents a unified framework for modeling the dry deposition of airborne MNP fibers by capturing the interplay of gravitational settling, Brownian diffusion, interception, and impaction. By bridging aerosol theory with fiber-specific corrections, the model reveals size- and shape-dependent behavior beyond gravitational settling. The results highlight the prolonged atmospheric residence times of submicron fibers and the strong spatiotemporal variability in deposition velocity, offering key insights for regional and global modeling of airborne plastic pollution. At the same time, this work represents an initial step toward coupling fiber dynamics with atmospheric aerosol frameworks. Important processes such as fiber stiffness, flexibility, deformation, and microstructural heterogeneity are not explicitly represented and may influence deposition under specific conditions. Incorporating these effects in ways compatible with large-scale atmospheric models remains an important direction for future experimental and mechanistic studies.

## Supplementary Material



## Notes

Detailed formulation of the dry deposition model for atmospheric micro- and nanoplastic fibers, including model formulation, uncertainty envelope analysis, data processing, computational pseudocode, and relevant references (PDF)
