# Supplementary material for: A Model for Dry Deposition of Atmospheric Micro- and Nanoplastic Fibers
Source: Environ Sci Technol Lett. 2026 Apr 29;13(5):737–43. doi: 10.1021/acs.estlett.6c00165 (PMC13173645; doi:10.1021/acs.estlett.6c00165)
Supplement: Supplementary file 1 [file ez6c00165_si_001.pdf]

Supporting Information

A Model for Dry Deposition of Atmospheric Micro- and Nanoplastic Fibers

Hosein Foroutan

Department of Civil and Environmental Engineering,
Virginia Tech, Blacksburg, Virginia 24061, United States
E-mail: hosein@vt.edu

Model formulation

This section summarizes the key equations used to estimate the total dry deposition velocity V_d of atmospheric micro- and nanoplastic (MNP) fibers, incorporating four processes: gravitational settling, Brownian diffusion, impaction, and interception.

The total dry deposition velocity is:

$$V_d = V_g + \frac{1}{R_a + R_s}$$

where V_g is the gravitational settling velocity.

The aerodynamic resistance R_a is calculated following a standard Monin–Obukhov similarity formulation (Zhang et al., 2001; Khan and Perlinger, 2017):

$$R_a = \frac{\ln\left(\frac{z_R}{z_0}\right) - \psi_H}{\kappa u_*}$$

where:

- z_R is the reference height (m),
- z_0 is the surface roughness length (m),
- $\kappa = 0.4$ is the von Kármán constant,
- u_* is the friction velocity (m s^{-1}),
- ψ_H is the stability correction function for heat (see, e.g., Khan and Perlinger, 2017).

The surface resistance R_s is defined as:

$$R_s = \frac{1}{\epsilon u_* (E_{\mathrm{br}} + E_{\mathrm{im}} + E_{\mathrm{in}}) R_1}$$

1. Gravitational Settling

The drag coefficient for cylindrical fibers is given by (Zhang & Choi, 2022):

$$C_d = \frac{58.58 \cdot \beta^{0.1936}}{Re^{0.8273}}, \quad \text{with} \quad Re = \frac{V_g d_{eq}}{\nu}$$

and

$$d_{eq} = \left(\frac{4l^2}{\beta\pi}\right)^{1/2}$$

Evaluation of the Reynolds numbers indicates that the present simulations largely remain within the nominal range of Zhang and Choi (2022), (1 < Re < 300) with Reynolds numbers falling below Re << 1 only at the smallest particle sizes. This regime corresponds to conditions where gravitational settling contributes negligibly to total deposition velocity, which is instead dominated by Brownian diffusion and interception. The settling formulation is therefore applied within its valid Reynolds-number range in the regimes where it controls deposition.

Gravitational settling velocity V_g is solved iteratively by finding the roots of this expression:

$$f(V_g) = V_g - \sqrt{\frac{4}{3}\left(\frac{\rho_p}{\rho_a} - 1\right) g \left(\frac{3l\pi}{8\beta}\right)\left(\frac{V_g\sqrt{4l^2/(\beta\pi)}}{\nu}\right)^{0.8273} \cdot \frac{1}{58.58\beta^{0.1936}}}$$

To assess whether the adopted settling formulation (Eq. (6) in the paper) produces realistic values, I compared it with more recent laboratory measurements of isolated microplastic fibers in air reported by Reininger et al. (2025). Using the reported fiber dimensions for their Samples 4–6 (see their Table 2), Eq. (6) yields settling velocities of approximately 0.10–0.12, 0.23–0.27, and 0.22–0.26 $\mathrm{m\,s^{-1}}$ for Samples 4–6, respectively, in close agreement with the measured values of 0.112, 0.246, and 0.248 $\mathrm{m\,s^{-1}}$. This comparison provides component-level corroboration of the shape-dependent settling parameterization.

Additionally, to examine the sensitivity of the settling component to particle material properties, we can vary the particle density ρ_p over a representative range for common polymer types (900–1400 $\mathrm{kg\,m^{-3}}$) while keeping all other parameters fixed. For a representative fiber of l=100 μm and β=10 the predicted settling velocity is 0.61, 0.67, and 0.89 $\mathrm{cm\,s^{-1}}$ for particle densities of 900, 1000, and 1400 $\mathrm{kg\,m^{-3}}$, respectively. This behavior reflects the expected dependence of gravitational settling on particle density.

2. Impaction

Stokes number:

$$St = \frac{V_g u_*}{gA}$$

where V_g is obtained from section 1.

Impaction efficiency:

$$E_{\mathrm{im}} = 0.4\left(\frac{St}{St + \alpha}\right)^{1.7}$$

The two parameters, α and A, are tabulated and vary with land use and season (see Table 3 in Zhang et al., 2001).

3. Brownian Diffusion

Schmidt number:

$$Sc = \frac{\nu}{D}$$

Diffusion coefficient:

$$D = \frac{k_B T C_c}{3\pi\mu d_{eq}}$$

Brownian collection efficiency:

$$E_{\mathrm{br}} = 0.2 \cdot Sc^{-2/3}$$

The diffusion formulation used here includes the standard Cunningham slip correction through C_c and therefore accounts for transition- and slip-flow effects for nanofiber-sized particles.

For fibers of diameter a and length l, with aspect ratio $\beta = l/a$, the equivalent diameter under isotropic Brownian motion is (Tian et al., 2016):

$$d_{eq} = \frac{a}{\frac{1}{k_{xx}} + \frac{1}{k_{yy}} + \frac{1}{k_{zz}}}$$

$$k_{xx} = k_{yy} = \frac{16(\beta^2 - 1)}{(2\beta^2 - 3)\ln\left(\frac{\beta + \sqrt{\beta^2-1}}{\sqrt{\beta^2-1}}\right) + \beta}$$

$$k_{zz} = \frac{8(\beta^2 - 1)}{(2\beta^2 - 1)\ln\left(\frac{\beta + \sqrt{\beta^2-1}}{\sqrt{\beta^2-1}}\right) - \beta}$$

4. Interception

Interception efficiency:

$$E_{\mathrm{in}} = 2.5\left(\frac{d_{eq}}{A}\right)^{0.8}$$

where equivalent diameter is calculated in section 3 and the constant A is from Table 3 in Zhang et al., 2001.

5. Total Dry Deposition Velocity

The total dry deposition velocity is then (Emerson et al., 2020):

$$V_d = V_g + \frac{1}{R_a + R_s}$$

where surface resistance is:

$$R_s = \frac{1}{\epsilon u_* (E_{\mathrm{br}} + E_{\mathrm{im}} + E_{\mathrm{in}}) R_1}$$

where:

- u_* is friction velocity,
- $R_1 = 1$ assumes no fiber bounce (a standard assumption for small, low-inertia particles in aerosol dry deposition models),

- $\epsilon = 3$ is a dimensionless empirical parameter (commonly adopted in resistance-based dry deposition parameterizations to maintain consistency with established aerosol models).

Uncertainty envelope for fiber dry deposition velocity

Figure S1 illustrates the dry deposition velocity of microplastic fibers as a function of fiber length for four representative land use types (broadleaf forest, grassland, water, and urban). The central curves correspond to the fiber-specific dry deposition parameterization developed in this study, which extends the resistance-based framework of Emerson et al. (2020) by incorporating shape-dependent settling and equivalent-diameter formulations for fibers. Shaded regions indicate a $\pm 5\times$ uncertainty envelope, consistent with the bounding approach adopted by Emerson et al. (2020), and reflect dominant uncertainties associated with surface resistance and collection parameterizations rather than uncertainties introduced by the fiber-shape corrections.

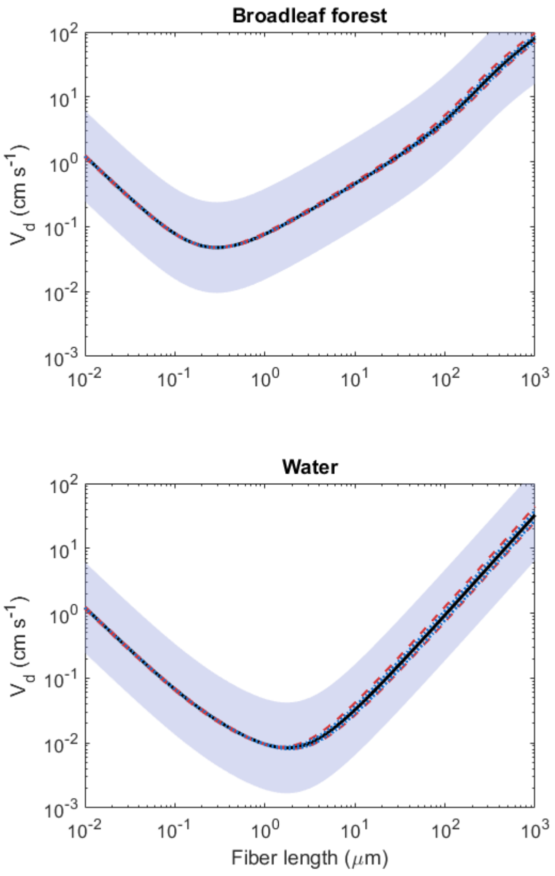

Figure S1: Dry deposition velocity of microplastic fibers as a function of fiber length for four representative land use types (broadleaf forest, grassland, water, and urban). The solid black line shows the baseline prediction, while the shaded region indicates a $\pm 5\times$ uncertainty envelope following the bounding approach of Emerson et al. (2020). Red dashed and blue dotted lines show sensitivity to fiber aspect ratio (β) and density (ρ_p), respectively, with $\pm 50\%$ variations relative to the baseline. These variations remain within the $\pm 5\times$ uncertainty envelope across all sizes and land-use types. The baseline case assumes a friction velocity u_* of 0.4 m s^{-1}, a plastic density ρ_p of 1200 kg m^{-3}, and an aspect ratio β of 10. Fibers are assumed not to bounce upon contact with the surface ($R_1 = 1$). Summer values of the land-use-dependent parameters α and A from Zhang et al. (2001) are used. Both axes are logarithmic.

Input datasets and processing

Friction velocity u_* was obtained from hourly, two-dimensional data collection `M2T1NXFLX` (`tavg1_2d_flx_Nx`) of the Modern-Era Retrospective Analysis for Research and Applications, Version 2 (MERRA-2), with a spatial resolution of $0.625° \times 0.5°$. PBLH was obtained from the corresponding monthly mean dataset `M2TMNXFLX` (`tavgM_2d_flx_Nx`), where PBLH is provided as a two-dimensional field at monthly temporal resolution. Land cover classification was based on the MODIS Terra+Aqua Land Cover Type Yearly L3 data product (MCD12C1) at a resolution of 0.05°. Each grid cell was assigned a land-use category, which was mapped to surface resistance parameters (α and A) following Zhang et al. (2001) (see Table S1).

Deposition velocity (V_d) was computed at hourly resolution for each grid cell using MERRA-2 u_* fields and land-cover-dependent parameters. For each time step, atmospheric lifetime was calculated as

$$\tau = \frac{\text{PBLH}}{V_d}$$

where PBLH corresponds to the monthly mean value for the given month. Monthly PBLH fields were applied to all hourly time steps within the corresponding month.

The resulting lifetime values were accumulated over all grid cells and time steps, and annual mean lifetimes were computed by averaging over all valid values.

The use of hourly u_* and monthly PBLH reflects their distinct temporal variability: u_* governs near-surface turbulent transfer and varies at sub-daily resolution, whereas PBLH represents the bulk mixing depth and evolves more gradually. As atmospheric lifetime is diagnosed from V_d rather than prognostically simulated, applying monthly PBLH provides a stable estimate without affecting the dominant variability in deposition velocity. Additional comparisons indicate that using monthly mean instead of hourly u_* introduces modest but noticeable quantitative differences in the predicted deposition velocity and atmospheric lifetime, while preserving the overall trends and relative differences across particle size and aspect ratio.

Air properties used in the deposition calculations were prescribed as constants. A representative temperature of $T = 298$ K was assumed, and dynamic viscosity, kinematic viscosity, and mean free path were computed accordingly. This choice isolates the influence of particle properties and deposition processes, and avoids introducing additional variability from meteorological fields.

Pseudocode for fiber dry deposition calculation

For each fiber length l and prescribed aspect ratio β:

1. Specify meteorological and air-property inputs, including friction velocity u_*, reference height z_R, roughness length z_0, Monin–Obukhov length L_O, air density ρ_a, dynamic viscosity μ, kinematic viscosity ν, temperature T, and fiber density ρ_p.

2. Select surface-specific parameters A and α according to the land-cover class using the tabulated values of Zhang et al. (2001). Table S1 shows how these values were mapped over MODIS land use types.

Table S1: Surface parameters α and A used for each MODIS land use type

MODIS land use type	Name	α (-)	A (mm)			
			Spring	Summer	Fall	Winter
0	Water bodies	100	1000	1000	1000	1000
1	Evergreen needleleaf forests	1.0	2	2	2	2
2	Evergreen broadleaf forests	0.6	5	5	5	5
3	Deciduous needleleaf forests	1.1	2	2	5	5
4	Deciduous broadleaf forests	0.8	5	5	10	10
5	Mixed forests	0.8	5	5	5	5
6	Closed shrublands	1.3	10	10	10	10
7	Open shrublands	50	1000	1000	1000	1000
8	Woody savannas	1.3	10	10	10	10
9	Savannas	1.3	10	10	10	10
10	Grasslands	1.2	2	2	5	5
11	Permanent wetlands	2.0	10	10	10	10
12	Croplands	1.2	2	2	5	5
13	Urban and built-up lands	1.5	10	10	10	10
14	Cropland/natural vegetation mosaics	1.2	2	2	5	5
15	Permanent snow and ice	50	1000	1000	1000	1000
16	Barren	50	1000	1000	1000	1000

3. Compute the gravitational settling velocity V_g of a cylindrical fiber by numerically solving the implicit drag-based formulation of Zhang and Choi (2022) (Eq. (6) in the main text). Here, the root of the nonlinear equation is obtained using MATLAB's `fzero` root-finding routine (Brent-type hybrid method, with the default tolerance set to machine precision).

4. Compute the Cunningham slip correction factor

$$C_c = 1 + \frac{2\lambda}{d}\left(1.257 + 0.4\exp\left[-0.55\,\frac{d}{\lambda}\right]\right),$$

   where λ is the mean free path of air.

5. Compute the equivalent diameter for Brownian diffusion using the isotropic mobility formulation of Tian et al. (2016):

$$d_{eq} = \frac{a}{\frac{1}{k_{xx}} + \frac{1}{k_{yy}} + \frac{1}{k_{zz}}},$$

   where a is the fiber diameter and k_{xx}, k_{yy}, and k_{zz} are functions of the aspect ratio β.

6. Compute the Brownian diffusion coefficient

$$D = \frac{k_B T C_c}{3\pi \mu d_{eq}},$$

and the Schmidt number

$$Sc = \frac{\nu}{D}.$$

7. Compute the Brownian collection efficiency

$$E_{\mathrm{br}} = 0.2\, Sc^{-2/3}.$$

8. Compute the Stokes number

$$St = \frac{V_g u_*}{gA}.$$

9. Compute the impaction efficiency

$$E_{\mathrm{im}} = 0.4 \left(\frac{St}{St + \alpha} \right)^{1.7}.$$

10. Compute the interception efficiency

$$E_{\mathrm{in}} = 2.5 \left(\frac{d_{eq}}{A} \right)^{0.8}.$$

11. Compute the surface resistance

$$R_s = \frac{1}{\epsilon u_* \left(E_{\mathrm{br}} + E_{\mathrm{in}} + E_{\mathrm{im}} \right) R_1}.$$

12. Compute the aerodynamic resistance (included here for completeness; in practice, this term is often obtained directly from the host model or existing aerosol modules).

$$x = \frac{z_R}{L_O}.$$

$$\psi_H = \begin{cases} 2 \ln \left(\frac{1+\sqrt{1-16x}}{2} \right), & -2 \le x < 0 \\ -5x, & 0 \le x \le 1 \end{cases}$$

$$R_a = \frac{\ln \left(\frac{z_R}{z_0} \right) - \psi_H}{\kappa u_*}.$$

13. Compute the total dry deposition velocity

$$V_d = V_g + \frac{1}{R_a + R_s}.$$

References

1. Emerson, E. W.; Hodshire, A. L.; DeBolt, H. M.; Bilsback, K. R.; Pierce, J. R.; McMeeking, G. R.; Farmer, D. K. Revisiting Particle Dry Deposition and Its Role in Radiative Effect Estimates. *Proc.*

Natl. Acad. Sci. U.S.A. **2020**, *117* (42), 26076–26082.

2. Tian, L.; Ahmadi, G.; Tu, J. Brownian Diffusion of Fibers. *Aerosol Sci. Technol.* **2016**, *50* (5), 474–486.

3. Zhang, J.; Choi, C. E. Improved Settling Velocity for Microplastic Fibers: A New Shape-Dependent Drag Model. *Environ. Sci. Technol.* **2022**, *56* (2), 962–973.

4. Zhang, L.; Gong, S.; Padro, J.; Barrie, L. A Size-Segregated Particle Dry Deposition Scheme for an Atmospheric Aerosol Module. *Atmos. Environ.* **2001**, *35* (3), 549–560.

5. Reininger, A.S.W., Tatsii, D., Bhowmick, T., Bagheri, G. and Stohl, A. The atmospheric settling of commercially sold microplastics. *Atmospheric Chemistry and Physics* **2025** *25* (18), 10691-10705.

6. Khan, T.R. and Perlinger, J.A., 2017. Evaluation of five dry particle deposition parameterizations for incorporation into atmospheric transport models *Geoscientific Model Development* **2017** *10* (10), 3861-3888.
